# Electrogastrography-Derived Mean Power Ratio as an Exploratory Objective Measure of Feeding Intolerance in Preterm Infants

**DOI:** 10.3390/bioengineering13030342

**Published:** 2026-03-15

**Authors:** Soheila Norasteh, Lindsay Roblyer, Rinarani Sanghavi, Hanli Liu, Eric B. Ortigoza

**Affiliations:** 1 Department of Bioengineering, University of Texas at Arlington, Arlington, TX 76019, USAhanli@uta.edu (H.L.); 2 Division of Neonatal-Perinatal Medicine, Department of Pediatrics, UT Southwestern Medical Center, Dallas, TX 75390, USA; 3 Division of Pediatric Gastroenterology, Department of Pediatrics, UT Southwestern Medical Center, Dallas, TX 75390, USA

**Keywords:** electrogastrography, feeding intolerance, preterm infants, neonates, power spectral density

## Abstract

Feeding intolerance (FI) is common in preterm infants and disrupts enteral nutrition. Because clinical signs of FI are nonspecific, objective biomarkers are needed. In this exploratory study, we evaluated whether electrogastrography (EGG) can distinguish infants with FI from those with no FI (NFI) based on their gastric response to feeding. For each infant, the first available weekly EGG recording (postnatal week 1 or, if unavailable, week 2), comprising two consecutive feeding cycles, was analyzed. Each recording included pre-, during-, and post-feed segments. Power spectral density (PSD) was computed over 0.5–9 cycles per minute (cpm) to derive baseline mean PSD (mPSD) and PSD ratios (PSDR) for during/pre- and post/pre-feeding (PSDR_Dur/Pre_, PSDR_Post/Pre_). Mean power ratios (mPR) were calculated across bradygastria, normogastria, and tachygastria frequency bands. Group differences were assessed using bootstrap resampling. Eighty-four infants were analyzed (75 NFI, 9 FI). Baseline mPSD values were comparable between the two groups. FI infants demonstrated lower PSDR_Dur/Pre_ values in the bradygastria and tachygastria bands, whereas normogastria responses were similar. No differences were observed in PSDR_Post/Pre_. EGG detected attenuated gastric activity specifically during feeding and not after feeding in infants with FI, supporting its potential as a non-invasive physiologic marker that warrants further validation.

## 1. Introduction

Feeding intolerance (FI) is a common complication in preterm infants and is reported in up to 75% of those with very low birth weight [1,2,3]. Clinically, FI is characterized by an inability to tolerate enteral feedings and is typically inferred from non-specific signs such as gastric residual volumes exceeding 50% of the previous feed, abdominal distention, emesis, or interruptions in the planned feeding schedule [4]. These features are widely cited across studies as defining attributes of FI; however, their interpretation remains largely subjective and varies considerably between clinicians. Importantly, FI is associated with delayed advancement of enteral nutrition, prolonged exposure to parenteral nutrition and antibiotics, longer hospitalization, and clinical overlap with early necrotizing enterocolitis (NEC), underscoring the need for objective physiologic assessment of gastrointestinal function [3,5]. Moreover, FI can arise from two fundamentally different mechanisms: developmental feeding intolerance (DFI), which reflects transient gastrointestinal immaturity and dysmotility, and pathological feeding intolerance (PFI), which results from conditions such as ileus due to sepsis, NEC, spontaneous intestinal perforation, or bowel obstruction [3]. Because the same nonspecific signs can be observed in both scenarios, DFI is frequently misinterpreted as PFI, leading to unnecessary interruption or delay of feeds in infants who may simply require maturation rather than intervention [3]. Current bedside assessments rely heavily on observation rather than direct measurement of gastric activity and, as a result, provide limited insight into the underlying physiological state. This motivates development of an objective and non-invasive method to quantify gastric function and to support future efforts to distinguish a benign developmental process from true pathology.

Electrogastrography (EGG) is a noninvasive technique that records gastric myoelectrical activity from abdominal surface electrodes [6,7], primarily capturing the slow-wave component [8] known as the gastric rhythm (GR). Because these signals are weak and often susceptible to contamination from respiration, motion, and nearby organ activity, spectral analysis is commonly used to extract informative features from the frequency domain [6,9,10]. Power spectral density (PSD) enables quantification of gastric rhythms across frequency bands: bradygastria (brady: 0.5 ≤ GR < 2 cpm), normogastria (normo: 2 ≤ GR < 4 cpm), and tachygastria (tachy: 4 ≤ GR < 9 cpm). Since its introduction in the 1920s, EGG has been applied in adults, children, and preterm infants under both physiological and pathological conditions [11,12]. Standardized parameters, such as dominant frequency, dominant power, power ratio (postprandial vs. preprandial baseline), and rhythm stability (% time in normogastria), have been widely used to characterize gastric myoelectrical activity in clinical and research settings [6].

In adults, EGG abnormalities have been reported in functional dyspepsia and gastroparesis, including increased dysrhythmic activity and blunted postprandial responses, and several studies have linked postprandial EGG changes to impaired gastric emptying [13,14]. However, EGG has not been adopted in routine adult clinical practice, largely due to limitations in signal quality, inter-session reproducibility, diagnostic specificity, and standardization across devices and protocols [9]. In contrast, neonatal recordings may mitigate some adult limitations because infants have a thinner abdominal wall and less subcutaneous fat, improving signal transmission and electrode placement consistency [6]. Pediatric and neonatal studies suggest that EGG-derived metrics reflect developmental maturation and feeding related modulation of gastric activity [15,16,17]. For example, stomach activity measured by EGG differs between healthy children and those with dyspepsia or reflux, with affected children demonstrating more irregular rhythms and weaker meal-related responses [18,19,20]. In newborns, Precioso et al. reported that clinically stable neonates across gestational ages exhibited predominantly normogastric rhythms, with limited feeding modulation, consistent with presence of slow-wave activity prior to robust nutrient responsiveness [15]. Chaudhari et al. showed that frequency-specific power spectral density (PSD) features during feeding vary with gestational age, supporting the concept that EGG can provide quantitative indices of gastric maturation in preterm infants [16].

Despite this foundation, prior neonatal EGG work has largely focused on developmental physiology rather than the specific clinical problem of FI, and there remains a need for analysis strategies that improve comparability across infants and emphasize feeding-evoked physiologic modulation [6,9,10,16]. Absolute EGG power and dominant-frequency-based measures can vary substantially across individuals and recording conditions, limiting inter-subject interpretability. Ratio-based spectral metrics that normalize each infant’s feeding response to their own baseline may reduce scaling effects related to impedance, electrode placement, or abdominal wall differences and may better isolate physiologically relevant modulation of gastric activity during feeding. Additionally, whereas many neonatal studies compare only pre- and post-feeding intervals, analysis of the during-feeding period may more directly capture active physiologic stimulation and thus improve sensitivity to impaired feeding responses [16].

Accordingly, we hypothesized that feeding-related modulation of gastric myoelectrical activity is altered in infants with FI compared with infants with no FI (NFI). The specific aim was to determine whether EGG-derived power spectral density ratios and mean power ratio metrics across pre-, during-, and post-feeding periods in the brady-, normo-, and tachygastria bands can quantitatively distinguish infants with FI from NFI.

## 2. Materials and Methods

### 2.1. Study Design and Participants

This exploratory, longitudinal, prospective cohort study was conducted in the neonatal intensive care units (NICUs) at Parkland Health and Hospital System and Children’s Health in Dallas, Texas, between 2017 and 2022. The study protocol was approved by the Institutional Review Board at UT Southwestern Medical Center (IRB# STU112015-071). Parental informed consent was obtained before enrollment. Participants underwent weekly EGG monitoring beginning within the first 14 postnatal days and continuing until 40 weeks postmenstrual age (PMA), hospital discharge, or death, whichever occurred first. For each infant, the first available weekly EGG recording (postnatal week 1 or, if unavailable, week 2), comprising two consecutive feeding cycles, was analyzed. We focused on the first available weekly EGG recording to standardize early postnatal physiologic assessment and avoid confounding from evolving clinical trajectories. Subsequent longitudinal recordings will be evaluated in future studies.

A total of 100 infants were enrolled. Of these, 84 infants (79 preterm and 5 term) met the study criteria and were included in the analysis. Participants were preterm infants < 34 weeks’ gestational age (GA) and term infants ≥ 37 weeks’ GA at birth. Term infants were included within the no feeding intolerance (NFI) group and were not analyzed as a separate cohort. Exclusion criteria were: known congenital or chromosomal disorders, significant clinical instability, major skin abnormalities precluding electrode placement, nil per os (NPO) status or continuous feeding at the time of measurement, and absence of an early EGG recording during week 1 or 2 of life. Data from participants were reported previously in a pilot study of gastrointestinal development [21,22]. The current study differs because it describes an innovative approach using analytical metrics instead of traditional EGG parameters to address a distinct hypothesis focusing on feeding intolerance.

#### Feeding Intolerance Cohorts

Infants were divided into two cohorts based on objective clinical outcome criteria, rather than non-specific bedside signs and symptoms. The no feeding intolerance (NFI) group (n = 75) met both criteria of sustained feeding tolerance: (1) achievement of full enteral feeds (≥120 mL/Kg/day) sustained for ≥5 days without parenteral (intravenous) nutrition and (2) growth velocity of ≥10 g/Kg/day from the 1st day of full enteral feeds until 34 weeks +6 days postmenstrual age. The feeding intolerance (FI) group (n = 9) met 0–1 of these criteria and/or developed gastrointestinal pathology (ileus due to sepsis, necrotizing enterocolitis Stage ≥ IIa, spontaneous intestinal perforation, or bowel obstruction). The FI group consisted of developmental FI (DFI, n = 5) and pathological FI (PFI, n = 4), though the primary analyses focused on FI versus NFI.

EGG features were not used in cohort assignment. EGG recordings analyzed in this study were obtained during the early postnatal period and were not temporally restricted to active FI episodes. Group classification therefore reflected overall feeding trajectory and clinical outcomes rather than contemporaneous symptoms at the time of recording. Gestational age, birth weight, feeding type, and other clinical factors were not used as matching variables or covariates in cohort assignment.

### 2.2. Experimental Setup

**Figure 1:**
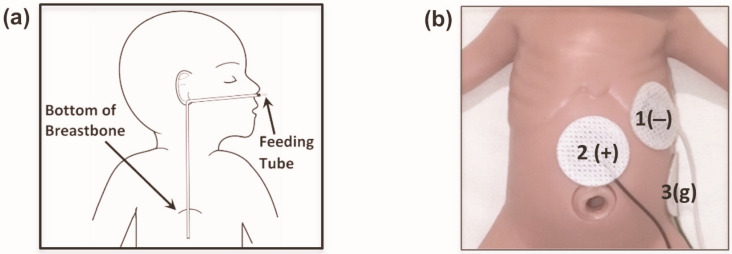
EGG Electrode Configuration [16]. (**a**) Typical configuration of an enteral feeding tube commonly employed in neonatal intensive care settings. (**b**) Arrangement of three EGG electrodes: The negative electrode (indicated by ‘1 (−)’) is positioned in the upper left quadrant, close to the mid-clavicular line. The positive electrode (marked as ‘2 (+)’) is situated midway between the base of the breastbone and the navel, slightly below the negative electrode’s level. The ground electrode (identified as ‘3(g)’) is positioned at the mid-axillary line, beneath the left rib margin.

Neonatal EGG electrodes were applied to the abdominal skin of each neonate as described in previous studies [6,22,23]. Because of limited abdominal surface area of neonates, the setup was confined to three electrodes (Figure 1). Prior to electrode placement, the abdominal skin was cleaned with Nuprep^®^ (Weaver and Company, Aurora, CO, USA) gel to lower the impedance. Conductive paste Ten20^®^ (Weaver and Company, Aurora, CO, USA) was applied between electrodes and the skin to allow transmittance of gastric electrical signals. Data acquisition was performed using BIOPAC^®^ MP36R System (BIOPAC^®^ Systems, Inc., Goleta, CA, USA).

### 2.3. EGG Acquisition

EGG recordings were conducted weekly and lasted approximately six hours to capture two full feeding cycles within a single recording. A feeding cycle is approximately 3 h long and it includes pre-feed, during-feed, and post-feed segments. Recording was started 30 min prior to the administration of the 1st feed and concluded 30 min prior to the administration of the 3rd feed. Feeding type (mother’s own milk, donor human milk, or formula) and feeding volumes were determined by the clinical team (blinded to EGG results) according to standard NICU protocols and were not controlled by the study.

### 2.4. Data Processing

The EGG data were processed following the procedures as briefly introduced below. This workflow encompassed signal preprocessing, segmentation into feeding-related periods, and subsequent spectral analysis, enabling consistent and reproducible evaluation of gastric myoelectrical activity across all participants.

#### 2.4.1. EGG Data Preprocessing

Raw EGG recordings were processed in MATLAB R2023b (MathWorks^®^, Natick, MA, USA) following a standardized pipeline. The data were first down sampled from 2000 Hz to 500 Hz to reduce computational load while preserving the frequency content of interest. A third-order polynomial fit was applied to obtain and then remove signal drift from the down sampled signal. The detrended signal was then low-pass filtered at 0.37 Hz (~22.2 cpm) using a zero-phase second-order Butterworth filter to attenuate high-frequency noise while avoiding phase distortion.

#### 2.4.2. Segmentation into Feeding Periods

Using recorded timestamps for the start and end of two feeding cycles per recording, the preprocessed EGG data were segmented into six distinct segments: pre-feed 1, during-feed 1, post-feed 1, pre-feed 2, during-feed 2, and post-feed 2, as described previously [16]. Briefly, the lengths of during-feed 1 and during-feed 2 were based on the actual duration of administration of the enteral feed. The lengths of pre-feed 1 and post-feed 2 were chosen to be ≤30 min. The lengths of post-feed 1 and pre-feed 2 were determined by calculating the time interval between the end of during-feed 1 and the start of during-feed 2, then dividing that time interval into two equal time-halves.

**Figure 2:**
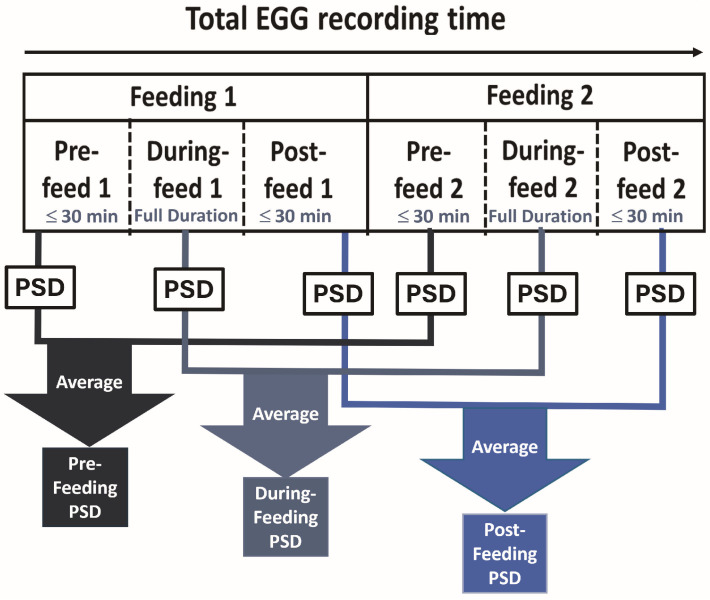
Schematic diagram showing how 6 distinct feeding segments were obtained from two feeding cycles. A PSD value was computed for each segment. PSDs from paired segments (from feeding 1 and feeding 2) were averaged to obtain 3 feeding phases (pre-feeding, during-feeding, and post-feeding PSD).

The first temporal half (up to 30 min) was labeled post-feed 1 and the second temporal half (up to 30 min) was labeled pre-feed 2. Only segments with at least 600 s (=10 min) of continuous data were included in the analysis. Thus, each recording included two pre-feed, two during-feed, and two post-feed segments, as labeled in Figure 2.

#### 2.4.3. Power Spectral Density (PSD) Calculation and Averaging Across Segments

For each of the six feeding segments, the PSD was computed over the gastric frequency band (0.5–9 cpm) using Welch’s method [24]. The 0.5–9 cpm range was selected to encompass the established bradygastria, normogastria, and tachygastria frequency bands. Processing was performed on signals down-sampled at 500 Hz and low-pass filtered at 0.37 Hz, with a 4 min window, 2 min overlap, and a resulting frequency resolution of 0.004 Hz (=0.24 cpm). The resulting PSD curves were averaged across paired segments to generate three combined feeding phases: (a) pre-feeding (average of pre-feed 1 and pre-feed 2), (b) during-feeding (average of during-feed 1 and during-feed 2), and (c) post-feeding (average of post-feed 1 and post-feed 2), as shown in Figure 2.

In addition, conventional EGG parameters were calculated as described previously [22] and are reported for descriptive comparison in Table A1 in Appendix A.

#### 2.4.4. Group-Level PSD Curves

For each infant, three PSD curves were generated corresponding to the pre-, during-, and post-feeding phases. These individual curves were then averaged within each group of (NFI, n = 75) and (FI, n = 9) to obtain group-level PSD profiles. This enabled direct comparison of gastric myoelectrical activity between FI and NFI groups at each feeding phase across all frequency ranges.

#### 2.4.5. Power Spectral Density Ratio (PSDR) Curves

For each infant, two PSD Ratio curves were generated by dividing the during-feeding PSD by the pre-feeding PSD (PSDR_Dur/Pre_) and the post-feeding PSD by the pre-feeding PSD (PSDR_Post/Pre_). These two ratio curves represent relative changes in gastric myoelectrical activity during and after feeding compared to baseline (pre-feeding). The PSDR_Dur/Pre_ curves were then averaged across all NFI infants (n = 75) and FI infants (n = 9), respectively. The PSDR_Post/Pre_ curves were averaged in the same manner for both groups.

### 2.5. Statistical Analysis

#### 2.5.1. Baseline Comparison

This analysis was exploratory/hypothesis-generating; no a priori power calculation was performed due to low FI prevalence in the cohort. Since our PSD power comparisons were based on ratios of PSDR_Dur/Pre_ or PSDR_Post/Pre_ between the two groups, we needed to verify that there were no pre-existing differences between EGG baseline (pre-feeding) power offsets of FI and NFI infant groups [25]. To facilitate statistical comparison of baseline gastric activity between the two groups, the pre-feeding PSD curves were used as the reference signals for respective groups. At the individual level, three mean PSD (mPSD) values were then obtained by averaging the PSD of pre-feeding within the three given gastric frequency bands: bradygastria, normogastria, and tachygastria, yielding mPSD_brady_, mPSD_normo_, and mPSD_tachy_.

At the group level, baseline gastric activity between the NFI (n = 75) and FI (n = 9) groups was compared using a nonparametric bootstrap resampling procedure with replacement (1000 iterations) to account for the unequal sample sizes [26,27,28]. This procedure was applied separately to each of the three baseline metrics (i.e., mPSD_brady_, mPSD_normo_, and mPSD_tachy_). For each iteration, an equal number of samples (n = group size) were drawn with replacement from each group, and the mean difference between groups was calculated for the metric of interest. Empirical 95% confidence intervals were defined by the 2.5th and 97.5th percentiles of the bootstrap distributions, and statistical significance was inferred when the interval excluded zero. Standardized effect sizes (Hedges’ g) were calculated for each comparison, and 95% confidence intervals were estimated from the bootstrap distribution. Hedges’ g was selected over Cohen’s d because it incorporates a small-sample correction factor, yielding less biased effect size estimates in cohorts with unequal and limited group sizes.

#### 2.5.2. Calculations and Comparisons of Mean Power Ratio (mPR) Metrics

After confirming no baseline differences in EGG between the two groups, mPR metrics were calculated for each infant by averaging PSDR_Dur/Pre_ and PSDR_Post/Pre_ values within the bradygastria, normogastria, and tachygastria bands. This yielded six metrics per infant: mPR_Dur/Pre_b_, mPR_Dur/Pre_n_, mPR_Dur/Pre_t_, mPR_Post/Pre_b_, mPR_Post/Pre_n_, and mPR_Post/Pre_t_ for both during- and post-feeding periods. Subscripts b, n, and t denote bradygastria, normogastria, and tachygastria frequency bands, respectively. To assess group differences, a non-parametric bootstrap resampling procedure (1000 iterations) was applied to each metric to generate the distributions of mean differences between the NFI and FI groups. Empirical 95% confidence intervals were defined by the 2.5th and 97.5th percentiles of these distributions, and statistical significance was determined when the interval excluded zero. In addition, standardized between-group effect sizes (Hedges’ g) were calculated, and corresponding 95% confidence intervals were estimated using the same bootstrap framework.

## 3. Results

### 3.1. Demographics of the Study Cohort

A total of 84 infants were included in the analysis, comprising NFI (n = 75) and FI (n = 9). Among the FI group, 5 were classified as DFI and 4 as PFI. Demographic and clinical characteristics for the cohort by feeding tolerance status are summarized in Table 1.

### 3.2. Group-Averaged PSD Curves Across Feeding Periods

**Figure 3:**
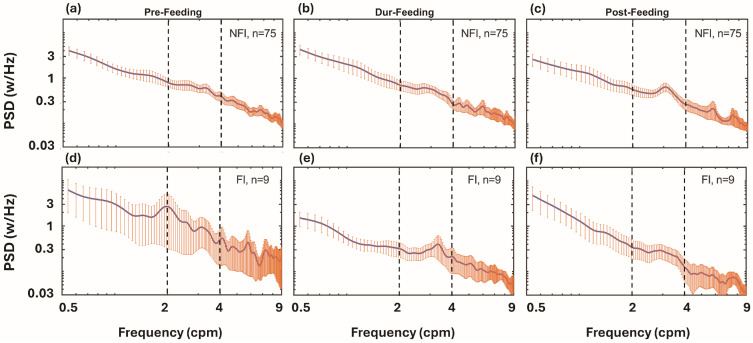
Group-averaged PSD curves for (**a**) pre-, (**b**) during-, and (**c**) post-feeding periods in NFI (n = 75) and (**d**–**f**) corresponding curves in FI (n = 9) infants. Vertical orange lines are the standard error of the means for each respective case. Vertical dashed lines separate the 3 frequency bands: bradygastria, normogastria, and tachygastria (from left to right).

After preprocessing, PSD curves were generated for the pre-, during-, and post-feeding periods of each infant. To illustrate overall gastric activity, PSDs were averaged within each feeding phase for the NFI (n = 75) and FI (n = 9) groups. Figure 3a–c displays the averaged PSD curves of the NFI group for the pre-, during-, and post-feeding phases, respectively. Figure 3d–f presents the corresponding curves of the FI group, allowing direct visual comparison between the two groups.

### 3.3. Group-Averaged PSD Ratio Curves with Respect to the Pre-Feeding PSD

**Figure 4:**
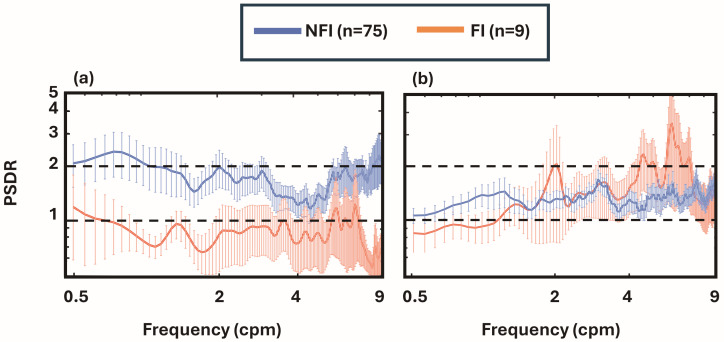
Group-averaged PSD (**a**) PSDR_Dur/Pre_ and (**b**) PSDR_Post/Pre_ for the NFI (n = 75; blue curve) and FI (n = 9; orange curve) groups. The vertical lines represent the standard error of the mean. Horizontal dashed lines represent the power ratios of 1 and 2.

To evaluate relative changes in gastric activity during and after feeding, PSD ratio curves were calculated for each infant by dividing the during-feeding and post-feeding PSD curves by the corresponding pre-feeding PSD. This yielded two ratio curves per infant: PSDR_Dur/Pre_ and PSDR_Post/Pre_. At the group level, these ratio curves were averaged within the NFI and FI groups, respectively, as shown in Figure 4. The resulting plots illustrate differences in both ratio magnitude and spectral distribution of feeding-related gastric activity between NFI and FI infants.

Visual inspection of the PSDR_Dur/Pre_ curves revealed a clear difference in feeding responses between the two groups. In the NFI group, most PSDR_Dur/Pre_ values ranged between 1.5 and 2 across 0.5–3 and 6–9 cpm, indicating a marked increase in gastric activity during feeding relative to baseline. By contrast, FI infants showed PSDR_Dur/Pre_ values consistently below or at 1, suggesting little to no modulation of gastric activity. This pattern indicates an attenuated or absent motility response in the FI group. However, the PSDR_Post/Pre_ curves showed no obvious separation between groups, suggesting that impaired gastric response in FI infants is most evident during, rather than after, feeding.

### 3.4. Baseline Gastric Activity: Bootstrapping Analysis of mPSD Values

**Figure 5:**
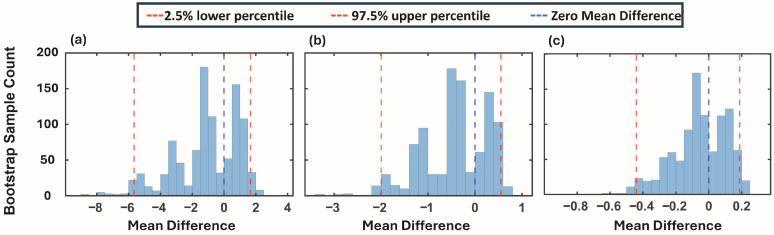
Distributions of bootstrapped mean differences in mPSD values for the pre-feeding period between the NFI (n = 75) and FI (n = 9) groups. Each panel (**a**–**c**) corresponds to one frequency band: (**a**) bradygastria, (**b**) normogastria, and (**c**) tachygastria. The x-axis shows the mean difference values, and the y-axis shows the bootstrap sample counts (visualized as histograms). The red dashed lines mark the 2.5th and 97.5th percentiles of the bootstrap distribution, while the blue dashed line indicates zero mean difference.

To assess baseline gastric activity, mPSD values for each frequency band—bradygastria, normogastria, and tachygastria—were calculated from the pre-feeding PSD curves of each infant. Bootstrapping with replacement was used to evaluate differences in baseline mPSD values between the NFI (n = 75) and FI (n = 9) groups. The bootstrapped distributions of mean differences indicated that the zero line fell within the 95% confidence intervals for all three frequency bands. Correspondingly, standardized effect sizes (Hedges’ g) were small and their 95% confidence intervals (CI) included zero across all frequency bands (bradygastria: g = −0.31; 95% CI −1.58–0.48; normogastria: g = −0.33; 95% CI −1.61–0.54; tachygastria: g = −0.17; 95% CI −1.19–0.49), consistent with the absence of significant baseline differences between groups (Figure 5a–c).

### 3.5. Gastric Activity Ratios: Bootstrapping Analysis of mPR Values

Since baseline mPSD values did not differ significantly between FI and NFI infants, relative changes in gastric activity during and after feeding were evaluated using mPR values. Accordingly, mPR ratios of mPR_Dur/Pre_ and mPR_Post/Pre_ were calculated within each of the three gastric frequency bands for each infant. This yielded six metrics per infant: mPR_Dur/Pre_b_, mPR_Dur/Pre_n_, and mPR_Dur/Pre_t_, computed by averaging PSDR_Dur/Pre_ within each respective band, and similarly mPR_Post/Pre_b_, mPR_Post/Pre_n_, and mPR_Post/Pre_t_ from PSDR_Post/Pre_.

**Figure 6:**
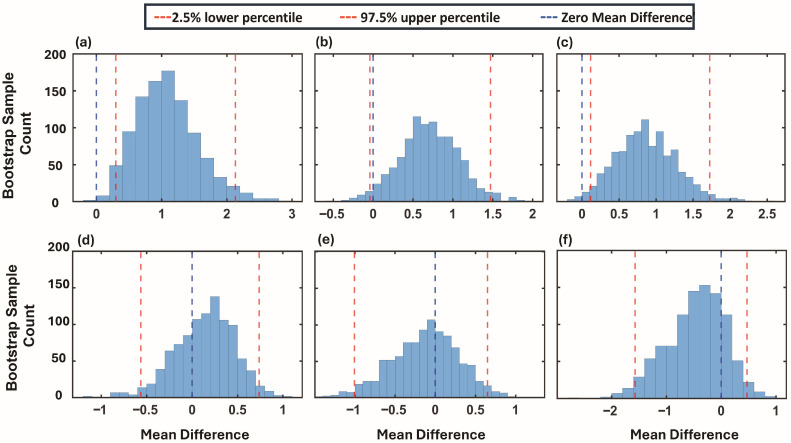
Bootstrapped distributions of mean differences in mPR values between NFI (n = 75) and FI (n = 9) groups for each gastric frequency band. Panels (**a**–**c**) show results for mPR_Dur/Pre_ metrics in the bradygastria, normogastria, and tachygastria bands, respectively. Panels (**d**–**f**) show results for mPR_Post/Pre_ metrics in the same frequency bands. The x-axis shows the mean difference values, and the y-axis shows the bootstrap sample counts (visualized as histograms). The red dashed lines mark the 2.5th and 97.5th percentiles of the bootstrap distribution, while the blue dashed line indicates zero mean difference.

Statistical differences between the NFI and FI groups were assessed using a nonparametric bootstrap resampling approach with replacement, applied separately to each metric. Results for the three mPR_Dur/Pre_ values are shown in Figure 6a–c, and those for the three mPR_Post/Pre_ values in Figure 6d–f.

Significant differences emerged for mPR_Dur/Pre_ in the bradygastria and tachygastria bands, indicating stronger feeding-related modulation in NFI infants. Standardized effect sizes (Hedges’ g) and confidence intervals (CI) were also calculated to quantify the magnitude of group differences. For mPR_Dur/Pre_, effect sizes were g = 0.29; (95% CI 0.14–0.48) for bradygastria and g = (0.31; 95% CI 0.08–0.57) for tachygastria, consistent with small-to-moderate group differences. In contrast, for the normogastria band, effect size confidence intervals included zero (g = 0.32; 95% CI 0–0.61), indicating no clear evidence of a between-group difference in this band.

Similarly, no significant differences were identified across mPR_Post/Pre_ metrics. Effect sizes for mPR_Post/Pre_ were small, and confidence intervals included zero across all frequency bands (bradygastria: g = 0.1; 95% CI −0.61–0.53; normogastria: g = −0.09; 95% CI −0.87–0.57; tachygastria: g = −0.34; 95% CI −1.31–0.33), consistent with the absence of significant post-feeding group differences. These findings suggest that group-related differences in gastric responsiveness were limited to the active feeding period and did not persist afterward.

For results reporting conventional EGG metrics see Table A1 in Appendix A.

## 4. Discussion

### 4.1. Key Findings of the Study

Electrogastrography is a noninvasive method that records gastric myoelectrical activity via abdominal surface electrodes, primarily capturing the gastric slow wave that regulates contraction rhythms [6,7,10]. Because raw EGG signals are weak and noisy in the time domain, frequency-domain spectral analysis is commonly used to characterize activity across three frequency bands: bradygastria (0.5–2 cpm), normogastria (2–4 cpm), and tachygastria (4–9 cpm) [9].

Building on this foundation, the present study applied a PSD-driven analysis to determine whether EGG signals can distinguish infants with and without FI based on their gastric myoelectrical responses to feeding. Gastric activity was evaluated using complementary spectral metrics (PSDs, PSD ratios, and mPR values) applied in a hierarchical manner. PSDs quantify how myoelectrical energy is distributed across frequencies, enabling assessment within the brady-, normo-, and tachygastria bands (Figure 3). PSD ratios were used to quantify feeding-induced changes relative to baseline. For example, a ratio ≤ 1 in PSDR_Dur/Pre_ or PSDR_Post/Pre_ (Figure 4) indicates minimal feeding-related spectral augmentation, consistent with reduced feeding-evoked gastric modulation. mPR metrics further summarize these ratio measures within each frequency band, providing compact numerical features for statistical comparisons between FI and NFI groups.

Group-averaged PSD ratio analysis showed that NFI infants exhibited higher PSDR_Dur/Pre_ values (~2) in the bradygastria and tachygastria bands, reflecting upregulation of gastric activity during feeding relative to pre-feeding baseline. In contrast, FI infants demonstrated blunted modulation, with PSDR_Dur/Pre_ values near 1, indicating reduced or absent feeding-associated spectral response. In comparison, no significant group differences were observed in PSDR_Post/Pre_ (post-feeding relative to pre-feeding), suggesting that the discriminatory physiologic signal is most evident during active feeding stimulation rather than in the post-feeding recovery phase. The post-feeding period may be more variable due to timing and physiologic state transitions.

Feeding induces postprandial augmentation of gastric electrical activity through enteric and vagal modulation of slow-wave activity [6,9]. In dysmotility states, post-feeding spectral modulation may be blunted despite preserved baseline rhythm [14]. After feeding ceases, gastric activity typically returns toward baseline levels [6], which may reduce between-group contrast in the post-feeding interval.

This pattern is consistent with prior pediatric and neonatal EGG studies demonstrating that feeding-related gastric modulation is developmentally variable and often attenuated in immature or dysmotile states, with preserved baseline slow-wave activity but reduced post-feeding augmentation [15]. In adult populations, functional dyspepsia and gastroparesis are associated with increased dysrhythmic activity and blunted post-prandial EGG power responses, supporting the interpretation that reduced feeding-evoked spectral modulation reflects disordered gastric motor control [14].

Mechanistically, normogastria reflects the dominant slow-wave rhythm and may remain relatively preserved, whereas bradygastria and tachygastria bands may be more sensitive to dysrhythmic or stress-related modulation [6,9,14]. This provides physiologic basis for the stronger group separation observed in the non-normogastric bands (bradygastria and tachygastria) in the present study. Overall, the findings support the study hypothesis that feeding-related modulation of gastric myoelectrical activity is altered in infants with FI compared with infants with NFI and that spectral power-based EGG analysis enables group-level discrimination between infants with and without FI based on feeding-related gastric myoelectrical responses.

### 4.2. Novel Analytical Development of the Study

EGG has seen limited adoption in routine adult clinical practice due to concerns regarding signal reproducibility, diagnostic specificity and cross-study standardization [9,29]. Surface recordings are affected by tissue thickness, adipose attenuation, and electrode placement variability, all of which can influence measured signal amplitude and spectral power [6]. In neonates, however, the thinner abdominal wall and reduced subcutaneous fat layer improve gastric signal transmission and electrode positioning consistency, which may enhance signal-to-noise ratio characteristics compared with adult recordings [17]. This physiologic difference supports continued investigation of EGG as a non-invasive gastric monitoring modality in preterm infants.

Conventional EGG metrics (Table A1 in Appendix A), such as absolute power, dominant frequency, or percentage of normogastria, are widely used but are known to vary substantially across individuals and recording conditions, which can limit inter-subject comparability [30]. In the present study, we therefore used ratio-based spectral metrics, including power spectral density ratio (PSDR) and mean power ratio (mPR), that normalize feeding responses to each infant’s own baseline. This within-subject normalization framework is designed to reduce the influence of amplitude scaling factors related to impedance, electrode coupling, and tissue transmission, and to emphasize relative feeding-evoked modulation rather than absolute signal magnitude [6,9]

Most prior neonatal EGG studies have focused on pre-feeding versus post-feeding comparisons. By contrast, our analysis explicitly incorporated the during-feeding interval, where physiologic stimulation is active and where group differences were most evident in the present dataset. This phase-specific ratio approach provides a complementary analytic perspective on feeding-evoked gastric modulation rather than relying solely on baseline-recovery contrasts [15,16].

In addition, we applied a non-parametric bootstrap resampling technique to compare groups with unequal sample sizes. Bootstrap methods provide distribution-free confidence interval estimation and are well established for inference under sample imbalance and non-Gaussian metric distributions [26,27,28]. This statistical design choice aligns with the exploratory, hypothesis-generating nature of this study and supports a more stable estimation of group differences in spectral ratio metrics without imposing parametric assumptions.

### 4.3. Clinical Implications of the Study

Results from this study suggest that attenuated feeding-related gastric myoelectrical responses may be associated with feeding intolerance. If validated, EGG may provide a non-invasive physiologic assessment approach to identify infants at risk for feeding intolerance, including pathologic forms such as necrotizing enterocolitis, which can lead to death or significant morbidity.

An objective physiologic measure may complement the current reliance on nonspecific clinical signs that often prompt interruption or delayed advancement of enteral feeds. Such interruptions may be unnecessary and may contribute to prolonged parenteral nutrition exposure, malnutrition, and poor neurodevelopmental outcomes in children who do not have FI [3]. If confirmed in larger cohorts, EGG-derived metrics could support future risk-stratification approaches.

Unlike radiographic or ultrasound studies, which provide structural or episodic functional snapshots, EGG offers continuous, bedside, radiation-free measurement of gastric myoelectrical activity and feeding-related physiologic modulation. Although the present research protocol required approximately six hours of recording to capture two feeding cycles, group differences were most evident during feeding, suggesting that shorter, targeted acquisition windows focused on pre- and during-feeding periods may be feasible in future translational workflows. EGG is not intended to replace imaging when bowel obstruction or surgical pathology is suspected, but rather to provide a complementary physiologic functional assessment.

### 4.4. Limitations and Future Work of the Study

This exploratory pilot study has several limitations. First, the number of preterm infants with FI was small, limiting statistical power. We therefore did not derive diagnostic thresholds, receiver operating characteristic (ROC) curves, or predictive performance metrics (e.g., sensitivity or specificity), as such analyses would be unstable with the limited number of FI cases. These evaluations will require larger, prospectively powered cohorts. Second, the temporal separation between Post-feed 1 and Pre-feed 2 segments was based on a fixed time window rather than physiological markers. Future studies should incorporate more precise methods to distinguish true postprandial recovery from subsequent baseline activity. Third, although Welch’s method was applied to reduce noise [16,24], EGG signals remain highly susceptible to motion artifacts. It should be noted that Welch’s method reduces variance in power spectral density estimates but does not explicitly remove motion-related artifacts arising from respiration, body movement, or abdominal muscle activity. As a result, residual motion contamination may have influenced the spectral content, particularly in higher-frequency bands such as tachygastria, where motion artifacts are more prominent. Accordingly, differences observed in these bands should therefore be interpreted with caution. No external motion sensors were used, and artifact removal was not explicitly performed prior to spectral analysis. Incorporating synchronized recordings of muscle activity (e.g., electromyography) or other movement-tracking sensors would allow more accurate identification and regression-based removal of motion artifacts. More advanced artifact rejection and denoising techniques will be essential to improve data quality in future work. Fourth, gestational age, birthweight, feeding composition (mother’s own milk, donor human milk, or formula) and administered volumes were not experimentally controlled and may contribute to between-infant variability in feeding-evoked gastric responses.

A critical next step is to move beyond FI versus NFI classification and determine whether EGG-derived spectral metrics can differentiate developmental (DFI) from pathological (PFI) feeding intolerance. These conditions reflect gastrointestinal immaturity versus true bowel dysfunction requiring distinct management strategies [3,23,31]. Although combined in the present analysis due to limited sample size, future studies with larger prospectively enriched cohorts may allow exploration of whether EGG-derived spectral signatures differ between these etiologies. It is plausible that PFI associated with structural or inflammatory pathology (e.g., necrotizing enterocolitis or septic ileus) could demonstrate more profound or persistent attenuation of feeding-evoked modulation compared with DFI, which primarily reflects transient gastrointestinal immaturity. Such distinctions remain speculative and will require adequately powered studies with stratified subgroup analysis. In addition, longitudinal modeling of repeated EGG recordings could clarify whether altered gastric modulation precedes, coincides with, or follows clinical FI episodes. Together, these directions represent important next steps toward more precise physiologic stratification.

## 5. Conclusions

This study demonstrates the feasibility of using EGG with ratio-based spectral metrics to quantify feeding-related gastric myoelectrical responses in preterm infants. Infants with feeding intolerance (FI) exhibited attenuated feeding-associated spectral power modulation compared with infants with no feeding intolerance (NFI), particularly within bradygastria and tachygastria frequency bands, while post-feeding measures were not significantly different between groups. The proposed normalization framework using power spectral density ratios and mean power ratios yields physiologically interpretable measures of feeding-related gastric response while reducing the influence of baseline signal scaling. These findings support the potential utility of normalized EGG spectral metrics as candidate objective indicators of feeding-related gastric response patterns; however, these results are exploratory and hypothesis-generating and require validation in larger, prospectively powered cohorts before clinical application.

## Figures and Tables

**Table 1:** Demographics.

Number (n) of Patients	NFI (n = 75)	DFI (n = 5)	PFI (n = 4)
Gestational Age (weeks)	29 [24–39]	30 [26–33]	27 [25–28]
Birthweight (g)	1391 [450–4245]	1350 [810–2000]	895 [710–1150]
Sex			
Female	41 [55%]	3 [60%]	2 [50%]
Male	34 [45%]	2 [40%]	2 [50%]
Race/Ethnicity			
Black Non-Hispanic/Latino	20 [27%]	3 [60%]	3 [75%]
White Non-Hispanic/Latino	2 [3%]	0 [0]	0 [0%]
Hispanic/Latino	50 [67%]	2 [40%]	1 [25%]
Asian	1 [1%]	0 [0]	0 [0]
Unknown/Not Reported	2 [3%]	0 [0]	0 [0]
Feeding Type			
Mother’s Own Milk (MoM)	45 [60%]	3 [60%]	4 [100%]
Donor Human Milk (DHM)	11 [15%]	1 [20%]	0 [0%]
Formula	18 [25%]	1 [20%]	0 [0%]
Mixture of MoM and DHM	0 [0%]	0 [0%]	0 [0%]
Mixture of MoM and Formula	1 [1%]	0 [0%]	0 [0%]
Feeding Volume (mL/Kg)	11.7 [1.6–20.4]	9.5 [2.5–15.4]	13.1 [9.1–17.2]
Age of First Feed (days)	2 [1–4]	2 [1–3]	2 [2–3]
Days of Hospitalization	68 [3–197]	62 [15–151]	90 [39–134]
Days on Parenteral Nutrition	11 [0–35]	10 [0–17]	60 [32–103]
Central Line Days	10 [0–38]	8 [0–13]	40 [8–105]
Days to Full Feeds	9 [2–35]	8 [6–9]	20 [6–34]
Days to Sustained Feeding Tolerance (SFT) *	10 [4–34]	8 [6–10]	25 [7–53]
Number of Times NPO	2 [0–10]	1 [0–3]	6 [3–14]
Number of Days NPO	2 [0–11]	1 [0–3]	16 [9–29]
Growth Velocity, g/Kg/day	15 [10–29]	9 [8–10]	5 [−11–14]
NEC Stage ≥ IIa			
Medical	0 [0%]	0 [0%]	1 [25%]
Surgical or Death	0 [0%]	0 [0%]	2 [50%]
Bowel Obstruction (Volvulus)	0 [0%]	0 [0%]	1 [25%]
Death	0 [0%]	0 [0%]	1 [25%]

Mean [Range] is presented for continuous variables and n [%] for categorical variables. Growth velocity from 1st day of sustained feeding tolerance to 34 weeks + 6 days postmenstrual age. * SFT = Sustained Feeding Tolerance (ability to achieve ≥120 mL/kg/day of full enteral feeds for ≥5 days without parenteral nutrition).

## Data Availability

The datasets generated during and/or analyzed during the current study are available from the corresponding author upon reasonable request and with appropriate institutional approvals. The data are not publicly available because the dataset is governed by institutional data-use policies requiring controlled access.

## Abbreviations

The following abbreviations are used in this manuscript:

EGG	Electrogastrography
FI	Feeding intolerance
NFI	No feeding intolerance
DFI	Developmental feeding intolerance
PFI	Pathological feeding intolerance
NEC	Necrotizing enterocolitis
GA	Gestational age
PMA	Postmenstrual age
NICU	Neonatal intensive care unit
IRB	Institutional Review Board
NPO	Nil per os (nothing by mouth)
SFT	Sustained feeding tolerance
GR	Gastric rhythm
PSD	Power spectral density
mPSD	Mean power spectral density
mPSD_brady_	Mean power spectral density in bradygastria
mPSD_normo_	Mean power spectral density in normogastria
mPSD_tachy_	Mean power spectral density in tachygastria
PSDR	Power spectral density ratio
PSDR_Dur/Pre_	During-feeding/pre-feeding power spectral density ratio
PSDR_Post/Pre_	Post-feeding/pre-feeding power spectral density ratio
mPR	Mean power ratio
mPR_Dur/Pre_	During-feeding/pre-feeding mean power ratio
mPR_Dur/Pre_b_	During-feeding/pre-feeding mean power ratio in bradygastria
mPR_Dur/Pre_n_	During-feeding/pre-feeding mean power ratio in normogastria
mPR_Dur/Pre_t_	During-feeding/pre-feeding mean power ratio in tachygastria
mPR_Post/Pre_	Post-feeding/pre-feeding mean power ratio
mPR_Post/Pre_b_	Post-feeding/pre-feeding mean power ratio in bradygastria
mPR_Post/Pre_n_	Post-feeding/pre-feeding mean power ratio in normogastria
mPR_Post/Pre_t_	Post-feeding/pre-feeding mean power ratio in tachygastria
cpm	Cycles per minute
Hz	Hertz
Kg	Kilogram
g	Grams
mL	Milliliters
CI	Confidence interval

## Appendix A

**Table A1:** Group Comparisons Utilizing Conventional EGG Metrics.

Conventional EGG Metrics	No Feeding Intolerance (NFI), n = 75	Feeding Intolerance (FI), n = 9	*p* Value *
% Bradygastria, <2 cpm			
Pre-prandial	23 (16–31)	24 (18–32)	0.36
Post-prandial	23 (14–28)	25(17–26)	0.61
% Normogastria, >2 to ≤4 cpm			
Pre-prandial	22 (16–28)	23 (18–26)	0.32
Post-prandial	22 (17–27)	23 (19–25)	0.89
% Tachygastria, >4 to ≤9 cpm			
Pre-prandial	40 (30–46)	41 (31–47)	0.90
Post-prandial	40 (36–45)	40 (38–45)	0.66
Dominant Frequency, cpm			
Pre-prandial	1.8 (0.6–5)	0.9 (0.6–3.6)	0.36
Post-prandial	1.5 (0.6–4.5)	1.2 (0.6–3.6)	0.35
Dominant power, mV^2^/Hz			
Pre-prandial	0.3 (0–0.6)	0 (0–1)	0.12
Post-prandial	0.1 (0.1–0.6)	0 (0–0.1)	0.27
Power ratio	1.9 (0.2–57)	2 (0.3–4.6)	0.72
Instability coefficient			
Pre-prandial	0.7 (0.4–0.9)	0.7 (0.5–0.9)	0.19
Post-prandial	0.6 (0.4–0.9)	0.7 (0.5–0.8)	0.63

Methods utilized to obtain these conventional EGG metrics were described in our prior work [22]. Median (range) is presented for continuous variables. * Mann–Whitney U Test.
